# Effects of Detritus on the Mosquito *Culex pipiens*: *Phragmites* and *Schedonorus (Festuca)* Invasion Affect Population Performance

**DOI:** 10.3390/ijerph16214118

**Published:** 2019-10-25

**Authors:** Paul T. Leisnham, Brandon Scott, Andrew H. Baldwin, Shannon L. LaDeau

**Affiliations:** 1 Department of Environmental Science and Technology, University of Maryland, College Park, MD 20742, USA; branscott33@gmail.com (B.S.); baldwin@umd.edu (A.H.B.); 2 Cary Institute of Ecosystem Studies, Millbrook, NY 12545, USA; ladeaus@caryinstitute.org

**Keywords:** *Culex pipiens*, Festuca, Juncus, Phragmites, stormwater, Typha, West Nile

## Abstract

Species interactions that influence the performance of the exotic mosquito *Culex pipiens* can have important effects on the transmission risk of West Nile virus (WNV). Invasive plants that alter the vegetation communities of ephemeral ground pools may facilitate or resist the spread of *C. pipiens* (L.) by altering allochthonous inputs of detritus in those pools. To test this hypothesis, we combined field surveys of roadside stormwater ditches with a laboratory microcosm experiment to examine relationships between *C. pipiens* performance and water quality in systems containing detritus from invasive *Phragmites australis* (Cav.) Trin. Ex Steud., introduced *Schedonorus* *arundinaceus* (Schreb.) Dumort., or native *Juncus effusus* L. or *Typha latifolia* L. In ditches, *C. pipiens* abundance was unrelated to detritus species but female *C. pipiens* were significantly larger from ditches with *S. arundinaceus* and smaller with *J. effusus*. Larger and smaller *C. pipiens* were also produced in microcosms provisioned with *S. arundinaceus* and *J. effusus,* respectively, yet the per capita rate of population of change did not vary. Larger females from habitats with *S. arundinaceus* were likely caused by faster decay rates of *S. arundinaceus* and resultant increases in microbial food, but lower survival as a result of fouling and higher tannin-lignin concentrations resulted in little changes to overall population performance. Larger female mosquitoes have been shown to have greater potential for transmitting arboviruses. Our findings suggest that changed community-level interactions from plant invasions in urban ephemeral ground pools can affect the fitness of *C.* pipiens and possibly increase WNV risk.

## 1. Introduction

As the coexistence of exotic species becomes more common, understanding how their interactions affect the success and impacts of biological invasions is increasingly important. Interactions among exotic species’ can be divided into two general categories based on how they affect invasion success. Exotic species can have facilitative effects on one another, broadly proposed as the invasion meltdown hypothesis (IMH), whereby positive interactions amongst invasive species lead to positive population-level feedback that intensifies the impacts of invasive species or facilitates secondary invasions [1]. Alternatively, exotic species can have inhibitory effects on other exotics that are already established in or are newly colonizing a system, by decreasing niche opportunities [2], altering the physical environment and nutrient fluxes, or impacting food webs [3]. 

Mosquitoes that use small ephemeral water bodies, such as stormwater structures (e.g., catch basins, ditches) or containers (e.g., tree holes, trash) for their developmental stages (eggs and larvae), provide excellent model systems to investigate the effects of interactions between exotic species on invasions. Small ephemeral habitats typically have low photosynthetic activity from plankton and algae, and the predominant resource for mosquito larvae consists of allochthonous detritus (mostly plant material) and associated microbes [4]. Density dependent competition is common in these habitats and can strongly negatively affect the production of adults [5,6,7,8,9]. Different species of detritus support different quantities (and possibly different species) of microbial food for mosquito larvae, which reduces resource competition and improves mosquito performance [10,11,12,13]. For example, larvae raised with animal detritus (e.g., dead insects) have higher survival and faster development than larvae raised with plant detritus [14,15,16,17]. Likewise, larvae raised with rapidly decaying plant detritus have similarly better larval performance than larvae raised with slow-decaying plant detritus [14,15,16,17]. In turn, differences in larval performance as a result of detritus type have been related to adult body size [13,14,18] and survival [17,19], while variation in available larval food resources more generally have also been related to fecundity [20,21,22], biting rate [23], and susceptibility to viral infection [24,25,26], all of which are expected to affect disease transmission at the individual and population scales [27,28,29,30,31,32]. Although resource competition is often the dominant ecological process regulating mosquito populations in ephemeral habitats, populations may also be regulated by abiotic factors, including litter-based toxins. Rapidly decaying plant detritus can increase toxicity from secondary metabolites, such as phenolics, lignin, and tannins [33,34], or due to microbial blooms that coat the water surface and prevent the larvae from breathing [11]. 

Invasive plants in ephemeral habitats have the potential to dramatically alter the performance of larval mosquitoes by altering either food resources or toxin concentrations. For example, inputs of native versus invasive litters have been shown to be important in mediating the effects of density dependence on the invasive mosquito *A. albopictus* in container systems [35]. Further, also in container habitats, leaf detritus from two invasive shrubs was shown to alleviate density dependent competition and promote higher adult emergence of *Culex pipiens* (L.) than detritus from native species, and this outcome appeared to be related to differences in associated bacterial communities [12]. Fewer studies have examined the effects of detritus type on larval mosquito performance in ground pool habitats, but at least one study has shown that the mowing of invasive *Phragmites australis* and *Typha* spp. around the perimeters of stormwater detention ditches dramatically increased abundances of larval mosquitoes in the ditches and adults infected with West Nile virus (WNV) in adjacent resident areas [36].

*C. pipiens* invaded the northern United States from Europe in the 16–17th centuries and has since established a wide geographical distribution across temperate regions in the country [37,38]. *C. pipiens* has spread into numerous other regions worldwide to now be distributed circumglobally [39]. Despite being generally well described in its established regions, *C. pipiens* continues to invade new areas [40]. *C. pipiens* obtains its blood meals primarily from birds but also opportunistically bites humans [41]. The species is a vector of St. Louis Encephalitis virus and WNV; thus, impacts on its distribution and abundance via interactions with other invasive species is of human health importance [42,43,44]. *C. pipiens* regularly inhabit highly ephemeral containers and ground pools, the latter of which include stormwater ponds, ditches, and wetland perimeters. These systems are small, discrete, and shaded habitats that usually lack vertebrate (e.g., fish, frogs) and invertebrate (e.g., beetles, damselflies) predators [5,9,45]. Such pools often persist for long enough periods of time to allow for the production of adult mosquitoes and can represent an important source of biting adults [5,46]. Detritus from plant species around the perimeter of these habitats is likely to affect the development of *C. pipiens* larvae and the production and fitness of biting adults, thereby influencing their regional abundance and disease transmission risk.

Common plant species that colonize ephemeral ground pools include both native species, such as *Juncus effusus* L., (soft rush) and *Typha latifolia* L. (broad-leaved cattail), and exotic invasive species *Phragmites australis*, (common reed) and *Schedonorus arundinaceus* (Schreb.) Dumort. (tall fescue). *J. effusus* is a slow spreading grass-like perennial that spreads primarily through seed dispersal, but can also spread vegetatively [47]. It is tolerant of diverse site conditions, including fresh waters and seasonal wetlands, and thrives in shallow waters [47]. *T. latifolia* is a perennial of the cattail family that has the ability to clone rapidly and spread by seed. This species is tolerant of fresh and brackish conditions and water level fluctuations [48]. *P. australis* is a species that historically could not be easily classified as native or introduced in North America due to a sudden increase in its spread on the continent. This spread, however, was shown to be due an invasive Eurasian genotype that had been introduced in the early part of the 19th century [49]. *P. australis* is a perennial reed that can spread rapidly by vegetative reproduction in addition to the production of seeds. It has a large biomass, enabling it to out-compete resident plant species through interference and exploitative competition [50,51,52]. The species is abundant in freshwater and brackish marsh habitats and has become increasingly common in disturbed sites, such as stormwater structures [49]. An introduced plant that is well adapted to disturbed areas, *S. arundinaceus* is a long-lived, deep-rooted, and perennial bunchgrass native to Europe [53]. This grass spreads most effectively through seeding but may also have short and slowly spreading rhizomes. It is common in damp grasslands and along riverbanks, has been planted as a forage grass as well as a means of erosion control and phytoremediation, and is regularly managed around stormwater structures and other ground depressions [53]. 

We examined the potential of invasive *P. australis* and *S. arundinaceus* and native *T. latifolia* and *J. effusus* at affecting performance of larval *C. pipiens* in ephemeral ground pool systems. We conducted a field survey of stormwater roadside ditches to identify the density and diversity of mosquito larvae in habitats that received detritus inputs of either of these four focal species. We also conducted a laboratory microcosm experiment assessing the water quality of systems containing different detritus inputs and the subsequent population performance of *C. pipiens* in those systems. Field observations indicated that detritus from *P. australis, T. latifolia,* and *J. effusus* were entirely from senescent litter while that of *S. arundinaceus* was always fresh clippings from regular mowing. We hypothesized that detritus type altered the population performance of *C. pipiens* by affecting food availability or habitat toxicity. Based on our hypothesis we predicted that, in the field, variation in mosquito densities among roadside stormwater ditches would be related their dominant vegetation and, in the laboratory experiment, detritus type would differentially affect density dependent competition of mosquito larvae or density independent toxicity.

## 2. Materials and Methods 

### 2.1. Field Survey

Mosquitoes and plant detritus was sampled from 16 roadside stormwater ditches in the Watts Branch watershed (lat. 39.225°, long. 76.867°), Maryland and Washington DC (USA) in August 2014. Watts Branch is a predominantly suburban tributary of the Anacostia River and is located approximately 160 km upstream of the Chesapeake Bay. Ditches were grouped by the dominant plant (>90%) that bordered their edge and gave allochthonous detritus input, with *S. arundinaceus*, *J. effusus*, *P. australis*, and *T. latifolia* representing four ditches each. No collected mosquitoes are from endangered species and all collection sites were on publicly accessible lands; thus, no field permits were required to collect them. Based on prior observations earlier in the summer, each ditch was known to retain small ephemeral pools of water, contain detritus, and hold larval mosquitoes, so that they collectively represented stormwater structures that would likely produce pestiferous and vector adults. Ditches with *P. australis*, *T. latifolia*, and *J. effusus* regularly received senesced leaf litter, with litter mainly accumulating around their perimeter. Ditches with *S. arundinaceus* received pulses of fresh clippings when they were maintained by local public works departments; thus, we surveyed these ditches 1–2 days after a mowing event to capture these additions. All ditches were at least 750 m apart to maintain spatial independence and none had been sprayed with herbicides or mosquitocides during 2014. Within each ditch, sampling was focused on a single randomly selected pool. Pools ranged in size but were no larger than 1.4 m^2^ and 399 L so that we could realistically collect their entire detritus contents (Table 1), and their size did not vary between detritus types (Area: F_3, 13_ = 0.9501, *p* = 0.4530; Volume: F_3, 13_ = 1.10, *p* = 0.3953).

From each ditch, mosquitoes were sampled by taking 10 dips with a standard 400 mL dipper around its perimeter to extract a total of 4 L water. A total of 24 pupae were collected in these samples, which were allowed to emerge into adults and identified to species level upon return to the laboratory. Eighteen adults were *C. pipiens* females, which were dried (45 °C, >48 hrs) and had their wing lengths measured as a reliable indicator of their body size. The remaining six adults were *Aedes vexans.* All larvae were stored in ethanol on return to the laboratory, and later enumerated and identified to species. Total plant litter was collected with a 150 µm 10” D-net and by hand. All pools included litter that was within the water (‘wet’ litter), which presumably supplied immediate resources to the aquatic environment, and litter piled on top of the water surface or at the immediate edge of the water that would likely provision the aquatic environment over time. We collected, dried (60 °C, >72 hrs), and weighed both litter types to estimate total standing litter per area (m^2^) and volume (L) and total wet standing litter per volume (L) for each pool. Mosquitoes were regularly collected in D-nets, but were not retained for enumeration because D-net sweeps were difficult to standardize in the ditches, separating mosquitoes from detritus was difficult, and the majority of specimens were highly damaged by preventing reliable identification. Litter from *P. australis*, *T. latifolia*, and *J. effusus* ditches almost entirely consisted of senesced litter and represents normal plant material available to mosquitoes during their summer developmental period. Litter from *S. arundinaceus* ditches was unidentifiable to species but we assumed it was from mowed *S. arundinaceus* from in and around the ditch. Litter samples from one *S. arundinaceus* and one *J. effusus* ditch were improperly collected and dried; therefore, these study ditches were removed from our analyses to give 14 total study sites (File S1).

### 2.2. Experimental Litter and Mosquitoes 

We collected senesced leaf litter of *P. australis*, *T. latifolia*, and *J. effusus*, and fresh clippings of *S. arundinaceus* from three sites on the University of Maryland campus, College Park, Maryland (USA) that were known to be free from any herbicide or mosquitocide applications. We dried all detritus (60 °C, >72 hrs) and assessed its carbon:nitrogen (C:N) ratio by randomly selecting five leaves and using a LECO CHN-2000 instrument (Leco Corporation, MI). The remaining detritus was cut into ~3 cm strips. All mosquitoes used in this study were F_1-_F_2_ larvae derived from *C. pipiens* colonies that had been established from larvae collected from ephemeral ground pools in College Park, Maryland. Larvae were raised to adulthood on lacalbumin (MP Biomedicals LLC, Solon, OH, USA) and adult females were blood-fed on anesthetized mice to produce eggs. Eggs were hatched in lacalbumin solution and then rinsed within 24 hrs of use. Anesthetization of mice was done under University of Maryland IACUC Protocol #R-09-10.

### 2.3. Laboratory Experiment

The experiment consisted of sixteen treatments. Each treatment contained one of the four detritus types, as follows: *S. arundinaceus*, *P. australis*, *T. latifolia*, and *J. effusus* and one of four (10, 20, 30, and 40) *C. pipiens* densities. There were seven replicates of each detritus type at the 10 density level, six replicates at the 20 density level, and three replicates at the 30 and 40 *C. pipiens* densities to yield 76 total experimental units. Experimental units consisted of 400 ml tri-pour cups with 360 ml of DI water and provisioned with 1.0 ± 0.01 g of detritus and 100 µL microbial inoculate of water from a stormwater ditch. The amount of detritus per L, mosquito density per L, and mosquito density per g detritus were all within the range of conditions observed in roadside stormwater ditches (Table 1). Cups were randomized and placed in an environmental chamber at 26 °C with a 14:10 (L:D) photoperiod. Due to egg availability, cups were entered into the experiment on four dates (blocks). For each cup, <24 hr old *C. pipiens* larvae were added after five days of being added into the chamber (experimental day 0). Cups were supplemented with 1.0 ± 0.01 g of detritus on day 20 to mimic regular food addition in field ditches and DI water was added when needed to maintain 360 ml. Pupae were removed daily and placed in individual vials to eclose. Adult sex and eclosion day were recorded. Dry mass and wing length was measured for each female. 

For each cup, the proportion of female survival to adulthood, the mean female mass, and the mean female development time were calculated. These fitness measures were used to estimate per capita rate of population change (λ′) [54], as follows:

λ′=exp[ln[(1/N0)∑xAxf(wx)]D+[∑xxAxf(wx)∑xAxf(wx)]]

where *N*_0_ is the initial number of females in the cohort (assumed to be 50%), *A_x_* is the number of females eclosing on day *x*, *w_x_* is the mean body size of females eclosing on day *x*, *f(w_x_)* is a function relating to fecundity to body size, and *D* is the time from adult ecolosion to reproduction, assumed to be 10 days for *C. pipiens* [38] (File S2). There is limited information on the fecundity to female body size relationship for *C. pipiens.* Almost all the information on this function is of an ecological biotype of *C. pipiens, C. pipiens* form *molestus* Forskal. *C. pipiens* f. *molestus* has a number of behavioral and physicological differences from *C. pipiens,* including the almost exclusive use of subterranean larval habitats and the ability to produce eggs without a vertebrate bloodmeal (autogeny) [38,55,56]. Egg rafts from autogenous species tend to have fewer eggs than those of anautogenous species (egg production requiring a bloodmeal), thus using a size-fecundity relationship based on *C. pipiens* f. *molestus* is undesirable for *C. pipiens* that would inhabit an above-ground stormwater ditch [38]. Therefore, we developed a size-fecundity relationship for *C. pipiens* by rearing larvae from the colony and rearing them to adulthood. As adults eclosed they were placed in 20 L nylon screen cages and within 5–10 days were fed to repletion from an anaesthetized mouse, then isolated in 600 mL containers with a 40 mL cup of water. Egg rafts were allowed to hatch and in <1 day larvae were counted. After oviposition, all females were killed, dissected, and the numbers of mature eggs (stages 4 and 5; [57]) in their ovaries were counted. Fecundity was calculated by adding laid and unlaid mature eggs. Wings of all females were removed and measured. A total of 55 females entered the experiment. Killing and dissecting females after the first gonotrophic cycle is consistent with most prior studies that have examined the fecundity of mosquitoes (e.g., [21]) (File S2). 

We measured detritus decay, microbial activity, and tannin-lignin concetration from a separate set of ninety-six cups without mosquitoes. Twenty-four cups were provisioned with 1.0 ± 0.01 g of one the four litter species and 100 µL microbial inoculate of pond water. Containers were randomized and placed in the environmental chamber. On experimental days 5, 9, 12, and 26, we destructively sampled six cups of each plant treatment. Cups were homogenized, and one 10 ml sample was extracted to test for tannin-lignins (mg/L) using a Hach spectrophotometer and its Hach tannin-lignin test kit (Hach, Loveland, CO, USA). A 1 mL sample was also extracted from five of the six cups containing each of the detritus types (80 total) to test microbial energy output (µwatts/ml), which is a measure of microbial activity, using a 4100 Multi-Cell Differential Scanning Calorimeter (Calorimetry Sciences Corp., Lindon, UT, USA). Readings were recorded after a 60 min equilibration period at 26 °C using sterile techniques and following procedures of past studies [58,59,60]. After water samples were taken, all detritus was removed from each cup using a 100-µm sieve, dried (60 °C, >72 hrs), and had their mass recorded to calculate percentage mass remaining (File S2). 

### 2.4. Statistical Analyses

We tested the effect of plant species on both total standing litter per habitat area and volume, wet litter per volume, total mosquito density, total mosquitoes per wet litter from study ditches, and adult female body size using linear models (PROC GLM, [61]) Each response variable was log_10_ transformed (+1 for microbial energy output) to meet assumptions of normality and homogeneity. Since we only had 18 adult females from all ditches, we pooled individuals from ditches within each dominant plant species. We analyzed *λ′* and its constituent fitness variables (proportion female survivorship, mean female mass, mean female development time), C:N ratio, and water quality variables (detritus decay rate, microbial production, and tannin-lignin) using linear models (PROC GLM, [61]). Numbers of *C. pipiens* and day were continuous variables, and detritus species and experiment entry date (block) were class variables in their respective models. We tested among detritus species for the quality of slopes of λ′ and each fitness parameter versus numbers of *C. pipiens* and water quality variables versus day. If detritus species altered the density dependent competition for food or temporal water conditions, we expected that slopes would differ among detritus types, yielding significant *C. pipiens* density × detritus species or day × detritus species interactions. Survivorship was arcsine square-root + 0.5 transformed, and microbial energy output, detritus mass, and tannin-lignin concentration were each log_10_ transformed (+1 for microbial energy output) to meet assumptions of normality and homogeneity of variances. The value λ′ did not meet the assumption of normality or homogeneity of variances despite transformations; thus, we tested for effects using a randomization test [62]. Since we ran models for multiple dependent variables on the same experimental units, we used a sequential Bonferroni adjustment for tests within each statistical model, with experimentwise α = 0.05. We tested for significant differences among detritus types using pairwise contrasts, with Tukey–Kramer adjustment for all possible comparisons within each analysis.

## 3. Results

### 3.1. Field Survey

We sampled a total of 835 larval mosquitoes from the 14 study ditches. *C. pipiens* was the most common species, constituting 69.9% (584) of all specimens, and found in all 14 ditches. *A. vexans* (22.6%, 189), *Culex restuans* (4.2%, 35), *Culex territans* (2.6%, 22), and *Aedes japonicus* (<1.0%, 5) represented the other mosquito species that were collected. Standing litter varied considerably among ditches (Table 1). There was a significant effect of plant species on total litter per area (df_3, 10_, F = 12.72, *p* = 0.0009) and volume (df_3, 10_, F = 8.59, *p* = 0.0040), although post-hoc pairwise tests only detected differences among individual plant species for litter per area, with *J. effusus* ditches having lower litter than all other plant species (*p*-values = 0.0007–0.0136; other *p*-values = 0.3213–0.9455). There were no differences in wet litter per volume (df_3, 10_, F = 2.61, *p* = 0.1097), mosquito density (df_3, 10_, F = 1.43, *p* = 0.2925), or mosquito abundance per detritus amount (df_3, 10_, F = 0.91, *p* = 0.4696). There was a significant effect of plant species on the adult body size of females that had been collected as pupae (n = 18, df_3, 14_, F = 31.35, *p* < 0.0001), with females being larger from ditches containing *S. arundinaceus* (*p*-values < 0.0001–0.0044) and smallest from ditches containing *J. effusus* (*p*-values <0.0001–0.0012), compared with all other plant species. There was no difference in the mean size of females from ditches containing *P. australis* and *T. latifolia* (*p* = 0.8310).

### 3.2. Laboratory Experiment

**Figure 1:**
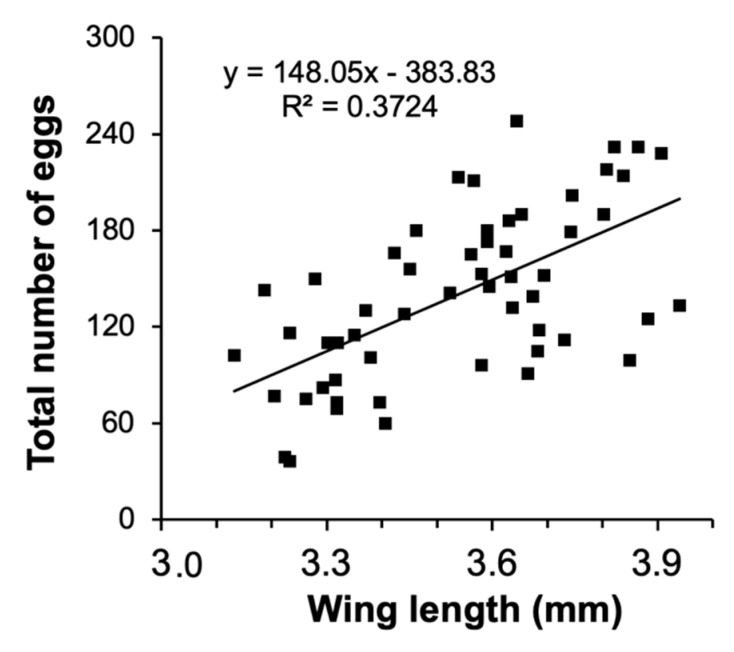
Relationship between number of eggs and wing length for *C. pipiens.*

**Figure 2:**
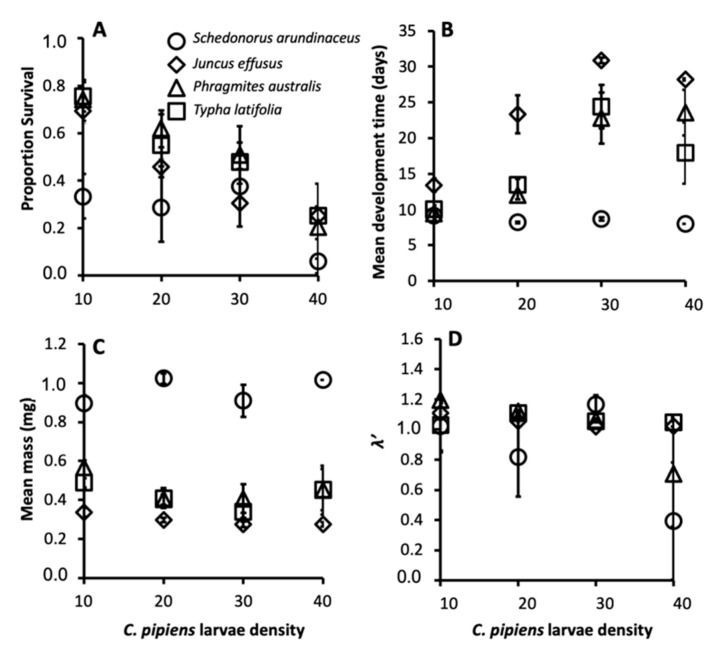
Mean female survivorship (**A**), female development time (**B**), female mass (**C**), and *λ′* (**D**) for *C. pipiens* provisioned with each detritus species. Error bars are ± Standard Error of the Mean (SEM).

The size-fecundity relationship we developed for *Culex pipiens* showed a significant positive relationship between female wing length and the number of eggs a female produced (r^2^ = 0.3724, n = 55; F_1,53_ = 31.45, *p* = 0.0001; Figure 1). Thus, we were able to use the size-fecundity relationship as the variable *f(w_x_)* in *λ′ (f(wx)*=0.5(148.05(*w_x_*) − 383.83)). Detritus litter, larval density, and their interaction did not affect *C. pipiens* λ′ but did affect individual fitness parameters (Table 2). *C. pipiens* survivorship was negatively affected by density and differed significantly among detritus species (Table 2). Survivorship in cups containing *S. arundinaceus* was significantly lower than in cups containing other detritus species (*p*-values = 0.0001–0.0100, Figure 2A). Female development time was longer in cups with higher larval densities but a significant density by detritus species interaction indicates that the density effect was moderated by detritus species (Table 2). Density significantly increased development time in cups with *T. latifolia, P. australis,* and *J. effusus* (*p*-values < 0.0001)*,* but had no effect in cups with *S. arundinaceus* (*p* = 0.4228) (Figure 2B). Female mass significantly differed among detritus species (Table 2), being significantly greater in cups containing *S. arundinaceus* than in cups containing other detritus species (*p*-values < 0.0001) and significantly less in cups containing *J. effusus* than in other detritus species (*p*-values < 0.0001–0.0014) (Figure 2C). 

**Figure 3:**
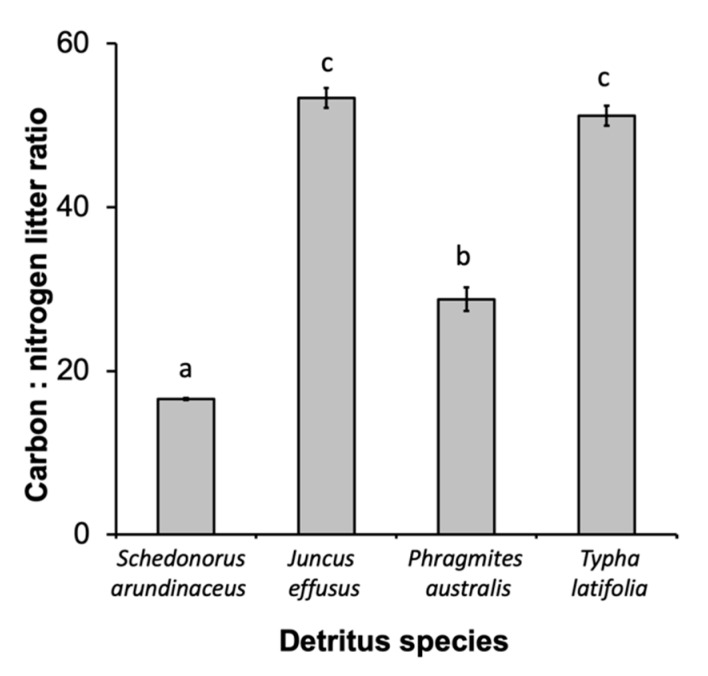
Carbon:nitrogen ratio for each detritus species. Different letters above bars denote statistically significant differences (experimentwise α = 0.05, sequential Bonferroni).

**Figure 4:**
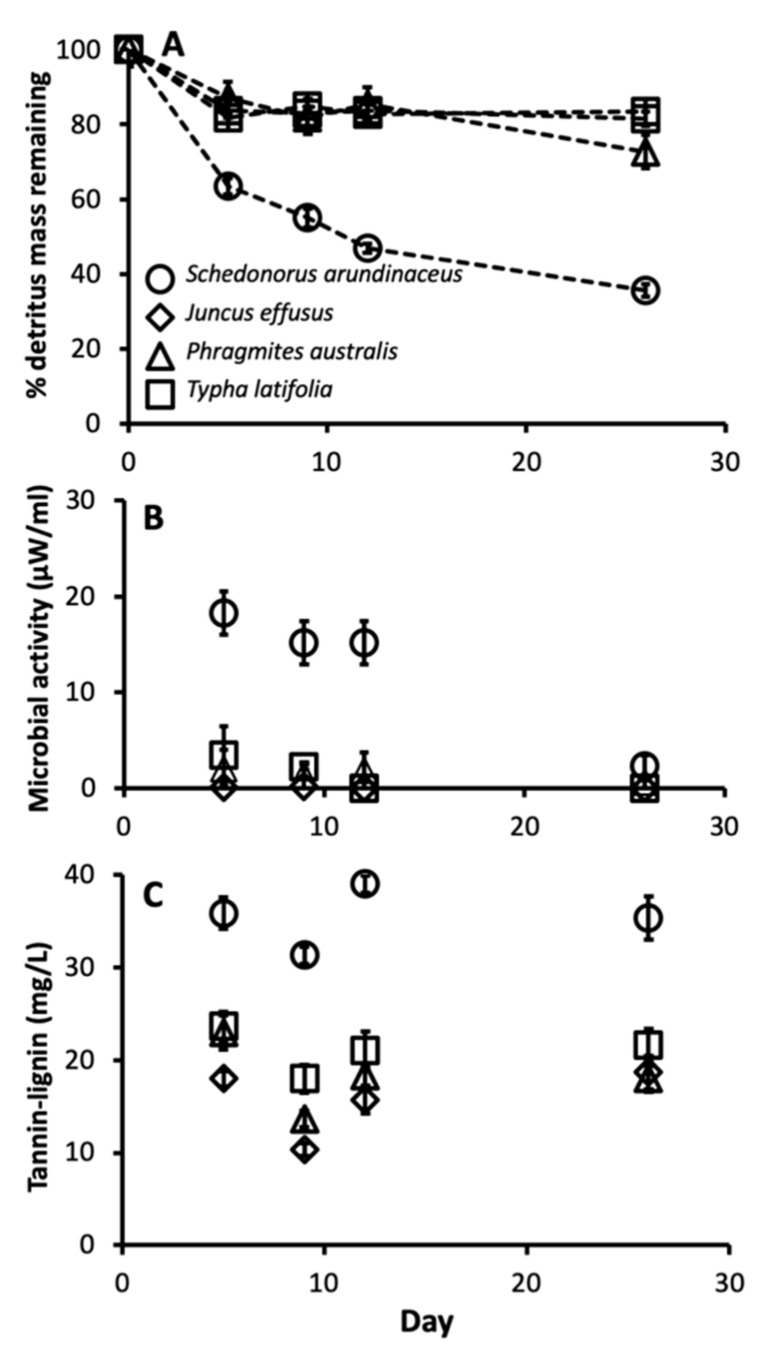
Mean microbial activity (**A**), % detritus mass remaining (**B**), and tannin-lignin concentration (**C**) over time for cups provisioned with each detritus species. Error bars are ± SEM.

There were also significant differences in leaf chemistry and water quality among different detritus species. The C:N ratio varied among different detritus species (F_3, 16_ = 253.35, *p* < 0.0001), with *J. effusus* and *T. latifolia* having the highest C:N ratios, followed by *P. australis* then *S. arundinaceus* with the lowest (Figure 3). Decay rate varied among detritus species, as indicated by a significant detritus by day interaction (Table 3). *S. arundinaceus* decayed much faster and ended the experiment with less % mass remaining compared to all other detritus species (*p*-values < 0.0001) whose decay rates did not vary among themselves (*p*-values > 0.10) (Figure 4A). Likewise, microbial activity was consistently higher in cups with *S. arundinaceus* than those with other detritus species over the duration of the experiment and declined at a faster rate than *J. effusus* (Figure 4B, Table 3). Tannin-lignin varied significantly by detritus species (Table 3), with concentrations being significantly greater in cups containing *S. arundinaceus* than all other detritus species and greater in cups containing *T. latifolia* than those containing *J. effusus* (*p* = 0.0001) and *P. australis* (*p* = 0.0080) (Figure 4C).

## 4. Discussion

The co-occurrence of exotic species is becoming more common with the increasing spread of organisms worldwide, and a better understanding of their interactions is important to manage biological invasions. Invasive plants that alter the vegetation around ephemeral ground pools may facilitate or resist the spread of the exotic vector mosquito *C. pipiens* by altering allochthonous detritus inputs in those pools. In surveys of roadside stormwater ditches, mean *C. pipiens* densities did not differ among habitats that varied in litter species, but female *C. pipiens* were larger from ditches with *S. arundinaceus* and smaller with *J. effusus* compared to other plant species (*P. australis* and *T. latifolia*). In a laboratory microcosm experiment, *C. pipiens* per capita rate of population change (λ′) was unaffected when larvae were provisioned with different detritus species, yet larger adults emerged from microcosms with *S. arundinaceus* and smaller adults from microcosms with *J. effusus*. For adults of numerous mosquito species, larger size is related to survival [63,64,65,66,67], and adult survival has been shown to be a critical life-history parameter for pathogen transmission in mathematical models [29,30,31] and field studies [67]. The results of this study suggest that the invasion of *S. arundinaceus* in stormwater ditches or the displacement of *J. effusus* by *P. australis* invasion may alter disease transmission of *C. pipiens* by producing larger females. Body size is also related to fecundity in mosquitoes [21,22,68,69]. As a part of this study, we developed a fecundity to female body size relationship for *C. pipiens* based on data from the wing lengths of 55 females. This allowed us to calculate more accurate λ′ estimates than past studies (e.g., [17,70]), which have used a function based on a relationship for four females from an autogenous form of *C. pipiens* that almost entirely uses subterranean environments, *C. pipiens* f. *molestus* [38,55,56]. Since egg rafts from autogenous mosquito species’ tend to have fewer eggs than those of anautogenous species, using a size-fecundity relationship based on a small number of *C. pipiens* f. *molestus* is undesirable for above ground *C. pipiens* [38]. Therefore, we recommend future studies that require size-fecundity relationship for above-ground *C. pipiens* to refer to the function in this study until an even more robust size-fecundity relationship from a greater number of females over a wider range of wing lengths is calulcated.

Our findings suggest that the litter species within ephemeral ground pools could affect the risk of disease transmission from *C. pipiens* in urban areas. Although mosquito size is a consistent factor explaining variability in vector competence, there are conflicting data in the literature as to whether smaller or larger female mosquitoes are more competent disease vectors. Evidence for the efficacy of smaller mosquitoes in transmitting disease was noted in a study on *A. albopictus* and *Aedes aegypti* susceptibility to the Sindbis virus [24]. This study demonstrated that smaller mosquitoes from environments with high density-dependent competition had a higher body titer and higher likelihood of infection than larger mosquitoes from low competition environments. Conversely, other studies have demonstrated that larger females are more efficient disease vectors [21,71,72]. Xue et al. found that larger *A. aegypti* females live longer and are more likely to seek multiple blood feedings than smaller females, which increased the likelihood that they could become infected with a virus and transmit it to a host within their lifetime [71]. Juliano et al. used both laboratory experiments and field-collected female *Ae. aegypti* to demonstrate that intraspecific competition in larval habitats produced smaller females that had reduced survival and were less likely to be infected with dengue [67]. If larger *C. pipiens* females are more effective disease vectors, ephemeral pool systems with *S. arundinaceus* clippings would produce the most competent disease-transmitting mosquitoes, while pools with *J. effusus* detritus with would produce the least competent disease vectors. Changes in vegetation communities surrounding ephemeral pools could then alter the risk of disease transmission from mosquitoes based on which plant species were displaced. For example, if *P. australis*, which produces intermediate-sized females, displaced *J. effusus* the disease risk in the area could increase, while if *P. australis* displaced *S. arundinaceus* the risk could decrease. Alternatively, if larger *C. pipiens* females are less efficient vectors, scenarios conducive to the establishment of *S. arundinaceus* would result in a lower risk of disease transmission. Future research is needed to examine the susceptibility of arboviral infection specifically for *C. pipiens* females at different sizes and from different larval environments. 

In this study, the effects of detritus species on *C. pipiens* performance in appear to be directly related to leaf chemistry and water quality. *S. arundinaceus* had the lowest C:N ratio, the highest decay rate, and supported the greatest microbial activity. Larger adults that emerged from microcosms with *S. arundinaceus* were likely exposed to much greater microbial food, while the higher nutrient concentrations fouled the water, as indicated by a thick microbial film that coated the water surface (*personal observation*), presumably causing lethally toxic conditions for a proportion of the larvae. These results are consistent with previous laboratory and field studies of the effects of detritus species and resultant food resources on mosquito performance (e.g., [10,11,15,73]), including those that have demonstrated increased survival and abundances from fresh compared to senescent detritus [14,36]. *S. arundinaceus* also leached higher tannin-lignin concentrations than other detritus species, reaching a maximum of 40 mg/L over the study duration. It is possible that these concentrations may have had a negative effect on *C. pipiens* performance. Nevertheless, they are substantially lower than concentrations reported to cause negative effects on other mosquito species in prior studies (e.g., >100 mg/L, [73,74]), and further work be needed to tease apart the effects of tannins and fouling on *C. pipiens* from *S. arundinaceus* and other litter species in ground pool habitats. Larval density also proved to be an important factor in survival and development of *C. pipiens*. Habitats containing fewer mosquitoes produced larvae that developed faster and had a higher rate of survival to adulthood, likely due to less competition for food resources. Based on the results of this study, it is likely that both density dependent and density independent processes influence the growth and survival of mosquitoes in ephemeral stormwater drainage ditches.

The overall effects of plant litter on mosquitoes can be broadly divided into two main categories, as follows: The chemical characteristics of the litter (i.e., nutrient availability, toxins) and the overall productivity of the species. We focused on the chemical characteristics of litter in our laboratory experiment by controlling the amount of litter that we added to the microcosms, and found that microcosms provisioned with *J. effusus* produced smaller females because of a slower decay rate and lower resource availability. We also recovered less litter from ditches with *J. effusus* compared to the three other plant species, indicating that the unfavorable conditions of *J. effusus* litter is likely compounded by its lower productivity in the field. These findings are consistent with past studies that appear to show much greater production from *P. australis* and *T. latifolia* than *J. effusus* [75,76]. Robust estimates of clippings from mowing *S. arundinaceus* or any other grasses in field habitats are scant but likely to be highly variable based on local management. Past studies have shown that plant clippings deposited from mowing stormwater BMPs (Best Management Practices) are often left in situ [77]. Based on the findings in our study here, we might expect the deposition of *S. arundinaceus* clippings from mowing to cause pulses of larger *C. pipiens* adults that are better disease vectors. To minimize disease risks from *C. pipiens,* local jurisdictions should consider capturing clippings around stormwater ditches and other BMPs that might provide habitat for this and vector mosquito species.

Few studies have compared the effects of invasive versus native litter on *C. pipiens* population dynamics [12,78], and only one study that we are aware of has addressed the effects of litter detritus on *C. pipiens* in ground pools [36]. MacKay et al. found that stormwater dry detention basins that received fresh clippings of invasive *Typha* spp. and *P. australis* that had been mowed had higher abundances of mosquitoes, including *C. pipiens*, than before mowing and to basins that were never mowed [36]. The authors concluded that, in addition to nutrient enrichment and increased larval survival, this result was also partly due to increased oviposition, based on an associated oviposition experiment that manipulated detritus additions in experimental microcosms. In our study here, we did not observe any relationships between detritus species on λ′ or survival in experimental microcosms in the laboratory or on densities in field ditches, suggesting that regional abundances of *C. pipiens* may be unaffected by the dominant detrital litter species. Body size is positively associated with mosquito fecundity [21,22,69], but greater egg production from larger females that have been raised on *S. arundinaceus* appear to be offset by decreases in larval survival, and likely explains why we observed no effects of litter species on abundances in field stormwater ditches. Nevertheless, we did not study oviposition rates in the field or experimentally test the effects of detritus species on *C. pipiens* oviposition and, therefore, its role on moderating relative abundances of the species among ditches surrounded by the plants in this study needs further study. Furthermore, *P. australis* and *S. arundinaceus* introductions could influence mosquito productivity through additional mechanisms other than altering resource availability, larval density, and fouling effects. Additional studies could examine the effect of *P. australis* and *S. arundinaceus* on nutrient removal from permanent stormwater BMPs, as well as interactions between detrital species, predation, and interspecific competition at the community level. Vegetation communities can also affect other aspects of adult mosquito ecology, such as resting sites, host habitat, or adult food sources; therefore, variability in the composition of local plant communities could indirectly affect oviposition in nearby ground pools by altering local adult abundances. Our field survey identified important relationships among plant species and *C. pipiens* in stormwater drainage ditches, but it was relatively limited in its scope, both with regards to space and time. Phenological differences among plant species (growth rates, nutrient sequestration, deposition of senescent litter) might alter the abundance and size of *C. pipiens* and other mosquito species developing in associated ground pool habitats. In particular, our conclusions on the effects of litter species on adult female body size and related pathogen transmission are based on only 18 individuals. Although we detected significant differences in adult size from ditches with different litter species, we only collected 2 females from ditches with *J. effusus* (6 from *S. arundinaceus* and *T. latifolia,* and 4 from *P. australis*). More extensive field and laboratory studies that test the effects of variable amounts and timing of detritus inputs on the performance of mosquito species among a wider range and greater number of sites would lead to a better understanding of vector ecology and associated transmission risks from stormwater BMPs. 

## 5. Conclusions

This study suggests that disease risks from *C. pipiens* may increase if the clippings from mowing introduced *S. arundinaceus* grass around stormwater drainage ditches or other BMPs are left in situ or if invasive *P. australis* displaces native *J. effusus.* Higher transmission risks appear to result from increases in microbial food that lead to larger females that likely have a greater potential for transmitting arboviruses. Our study is among the first to demonstrate that shifting community-level interactions from plant invasions in an ephemeral ground pool system might affect the fitness of *C. pipiens* and increase risks from West Nile virus. 

## Figures and Tables

**Table 1:** Mean ± SE (range in parentheses) litter and mosquito parameters in roadside ditches with either *S. arundinaceus, P. australis, T. latifolia,* or *J. effusus* as the dominant emergent plant species. All litter was dried before weighing.

Plant Species	Number of Sites	Area (m^2^)	Litter Per Area (g/m^2^)	Estimated Volume (L)	Total Litter Per Volume (g/L)	Wet Litter Per Volume (g/L)	Mosquitoes/Volume (no./L)	Mosquitoes/Wet Litter (no./g)
*S. arundinaceus*	3	0.56 ± 0.12 (0.40–0.80)	556.1 ± 198.6 (236.3–920.0)	169.3 ± 34.3 (104.0–220.0)	1.91 ± 0.82 (1.02–3.54)	1.63 ± 0.84 (0.66–3.31)	10.7 ± 4.1 (4.0–18.0)	7.5 ± 1.8 (5.43–11.00)
*P. australis*	4	0.95 ± 0.20 (0.40–1.40)	846.3 ± 73.1 (659.3–999.0)	279.0 ± 54.7 (148.0–378.0)	2.88 ± 0.49 (2.26–4.34)	1.19 ± 0.21 (0.74–1.61)	22.3 ± 5.9 (7.0–33.3)	18.5 ± 3.9 (7.4–25.8)
*T. latifolia*	4	0.66 ± 0.22 (0.32–1.30)	602.5 ± 109.1 (320.0–830.0)	206.9 ± 69.6 (92.0–390.0)	2.05 ± 0.56 (1.07–3.61)	1.40 ± 0.55 (0.47–3.00)	15.3 ± 3.2 (8.0–22.0)	19.1 ± 8.3 (2.7–40.7)
*J. effusus*	3	0.56 ± 0.12 (0.40–0.80)	136.9 ± 12.5 (123.8–162.0)	151.3 ± 23.1 (112.0–192.0)	0.50 ± 0.02 (0.45–0.54)	0.39 ± 0.01 (0.37–0.40)	8.8 ± 2.9 (3.0–12.5)	22.1 ± 7.2 (8.0–31.6)
Total	14	0.70 ± 0.10 (0.32–1.40)	562.4 ± 85.1 (123.8–999.0)	207.5 ± 27.6 (92.0–390.0)	1.92 ± 0.33 (0.45–4.34)	1.18 ± 0.25 (0.37–3.31)	14.9 ± 2.40 (3.0–33.3)	17.1 ± 3.1 (2.70–40.7)

**Table 2:** Results of randomization tests for λ′, and linear models for female survivorship (arcsine square-root transformed), mean female mass, and mean female development time for *C. pipiens*. Effects significant at experimentwise α = 0.05 (sequential Bonferroni) are shown in bold. Models included block (df = 3).

Variable	λ′	Survivorship			Mass			Development Time		
	Pr > F	df	F Value	Pr > F	df	F Value	Pr > F	df	F Value	Pr > F
**Detritus**	0.4190	3	1.43	0.2412	3	17.05	**<0.0001**	3	1.94	0.1332
**Density**	0.2330	1	13.06	**0.0006**	1	2.40	0.1270	1	69.88	**<0.0001**
**Density x Detritus**	0.2310	3	0.30	0.8256	3	1.54	0.2137	3	8.98	**<0.0001**
**Error**		65			58			58		

**Table 3:** Results from linear models for detritus decay rate, microbial activity (µwatts/mL), and tannin-lignin concentration (mg/L) (all log_10_ transformed). Effects significant at experimentwise α = 0.05 (sequential Bonferroni) are shown in bold.

Variable	Detritus decay			Microbial Activity			Tannin-Lignin		
	df	F Value	Pr > F	df	F Value	Pr > F	df	F Value	Pr > F
**Detritus**	3	716.15	**<0.0001**	3	41.31	**<0.0001**	3	72.91	**<0.0001**
**Day**	1	221.94	**<0.0001**	1	39.67	**<0.0001**	1	1.68	0.1980
**Day x Detritus**	3	36.45	**<0.0001**	3	3.90	0.0122	3	0.76	0.5213
**Error**	88			72			88		

## Supplementary Materials

The following are available online at https://www.mdpi.com/1660-4601/16/21/4118/s1, File S1: Field data, File S2: Lab data.
